# Supplementary material for: Interface Structure and Band Alignment of CZTS/CdS Heterojunction: An Experimental and First-Principles DFT Investigation
Source: Materials (Basel). 2019 Dec 5;12(24):4040. doi: 10.3390/ma12244040 (PMC6947346; doi:10.3390/ma12244040)
Supplement: Supplementary file 1 [file materials-12-04040-s001.pdf]

materials

Interface Structure and Band Alignment of CZTS/CdS Heterojunction: An Experimental and First-Principles DFT Investigation

Sachin Rondiya [1], Yogesh Jadhav [2], Mamta Nasane [3], Sandesh Jadkar [3] and Nelson Y. Dzade [1,*]

1 The School of Chemistry, Cardiff University, Cardiff, CF10 3AT, Wales Postcode, UK; RondiyaS@cardiff.ac.uk
2 National Center for Nanoscience and Nanotechnology, Mumbai University, Mumbai 400098, India; nano4yash@gmail.com
3 Department of Physics, Savitribai Phule Pune University, Pune 411007, India; nasanemamta@gmail.com (M.N.); sandeshjadkar@gmail.com (S.J.)
* Correspondence: DzadeNY@cardiff.ac.uk

The supplementary Information contains the crystal structure, band structures, partial density of states (PDOS) of stannite-CZTS and CdS. The geometry optimized model of the CdS(100)/st-CZTS(001) interface and the corresponding electrostatic potentials across the interface are provided.

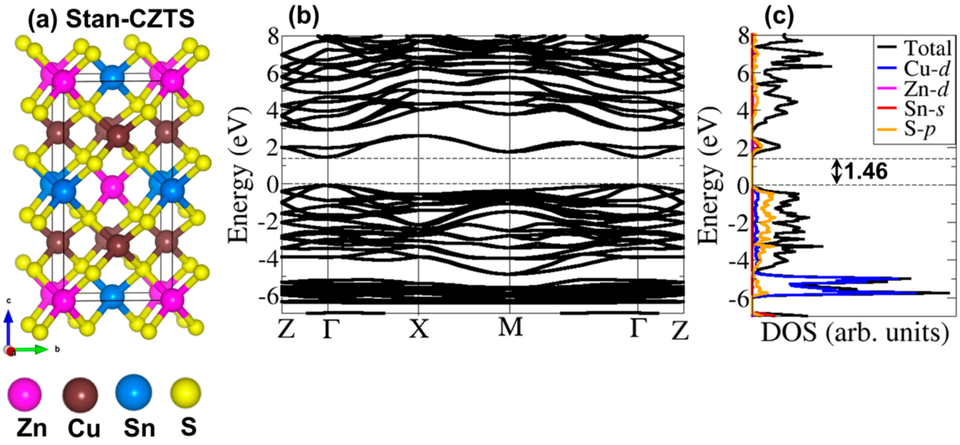

Figure 1. (**a**) Crystal structure, (**b**) band structure along the high-symmetry directions of the Brillouin zone, and (**c**) partial density of states (PDOS) of stannite-CZTS.

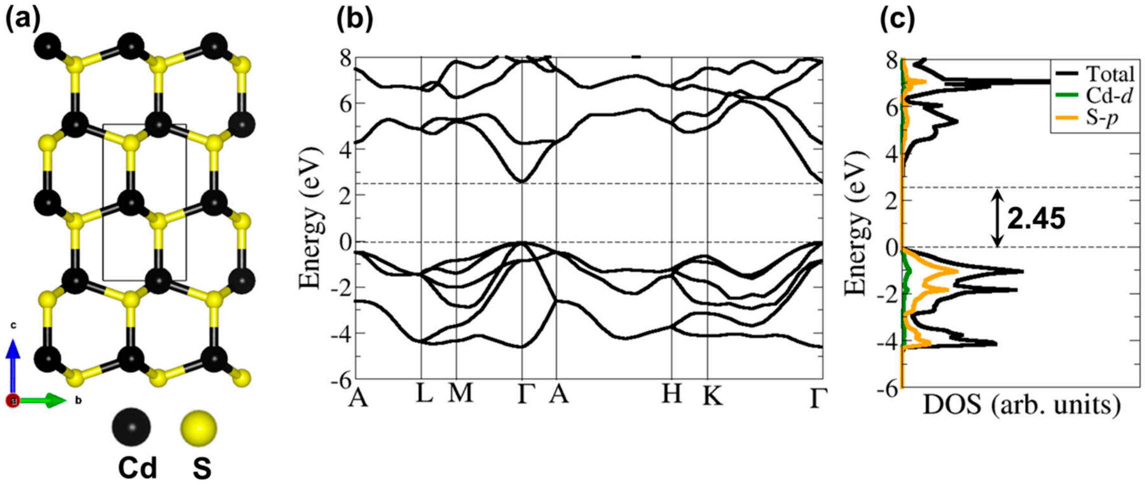

Figure 2. (**a**) Crystal structure of the hexagonal CdS. (**b**) Band structure along the high-symmetry directions of the Brillouin zone of CdS. (**c**) The density of states (DOS) of CdS.

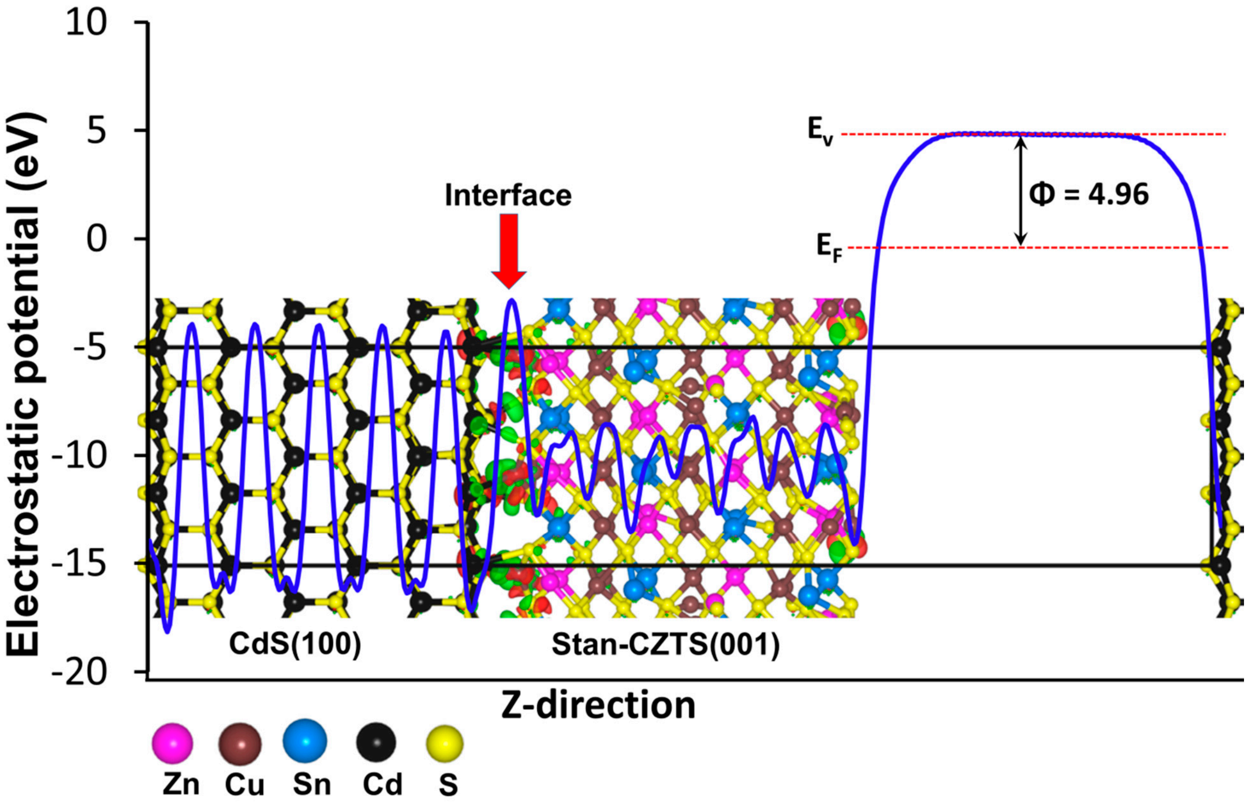

Figure 3. Geometry optimized model of the CdS(100)/st-CZTS(001) interface and the corresponding electrostatic potential (solid blue line), with the vacuum (E_V) and Fermi (E_F) level indicated by broken red line. Φ is the work function.

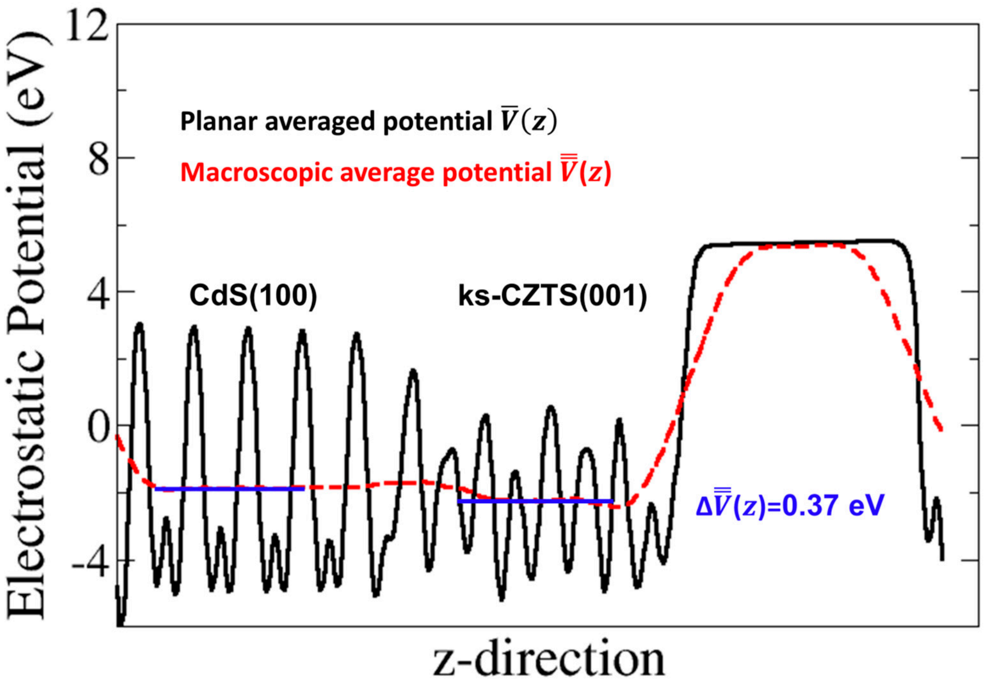

Figure 4. Electrostatic potential profile for the CdS/ks-CZTS heterojunction along the [001] direction. The blue solid line represents the macroscopic average of the electrostatic potential across the interface and ΔV stands for the resulting lineup.

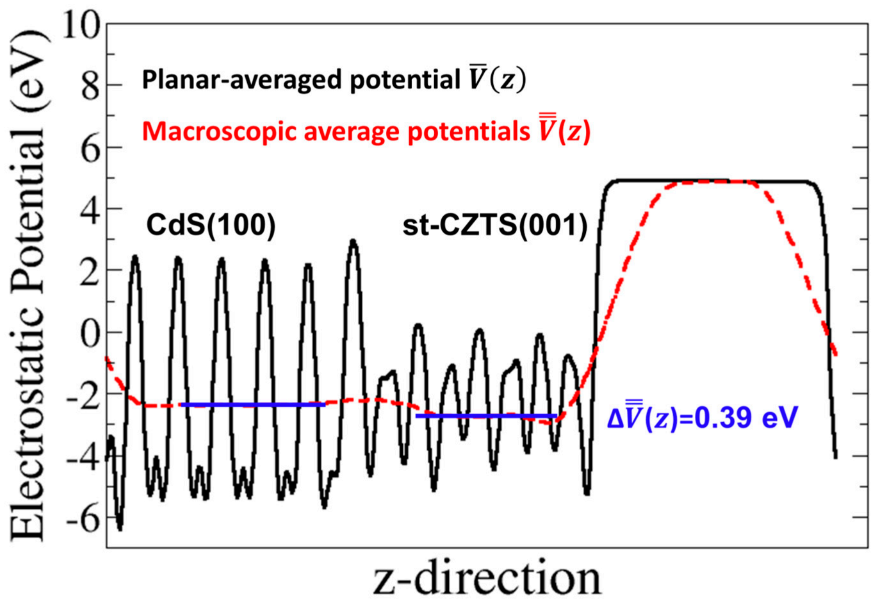

Figure 5. Electrostatic potential profile for the CdS/st-CZTS heterojunction along the [001] direction. The blue solid line represents the macroscopic average of the electrostatic potential across the interface and ΔV stands for the resulting lineup.

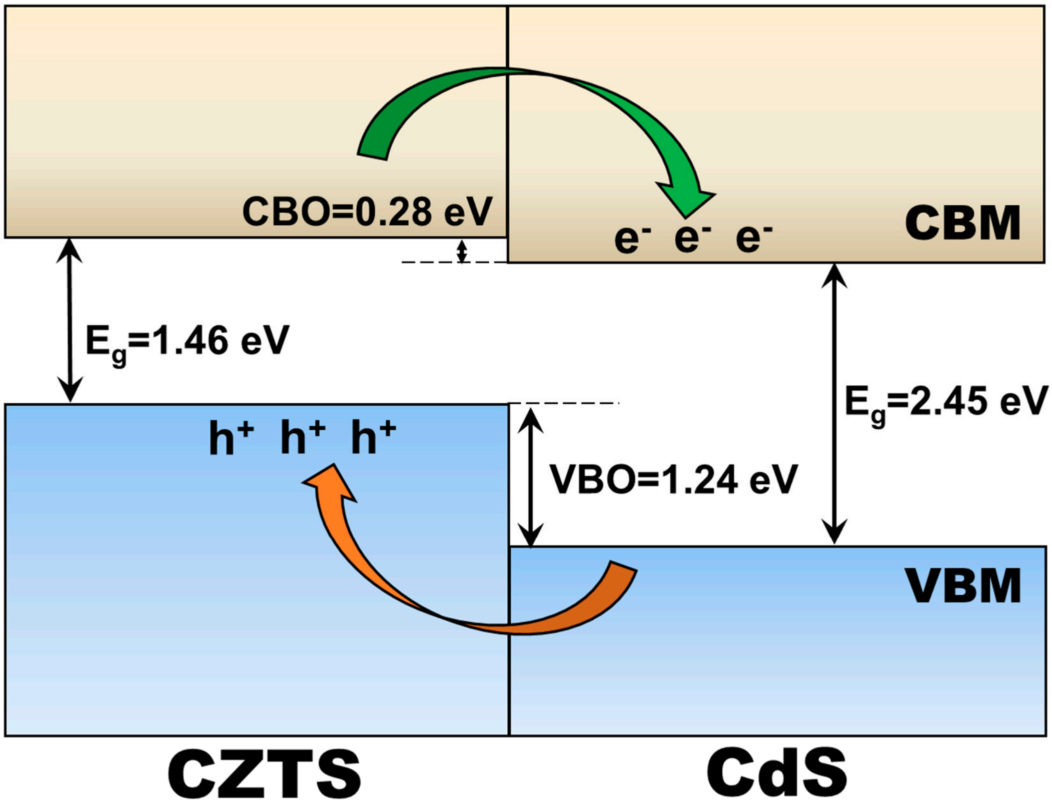

Figure 6. DFT predicted energy band alignment diagram of the CdS/st-CZTS heterojunction.
